# Decreased Placental FPR2 in Early Pregnancies That Later Developed Small-For-Gestation Age: A Potential Role of FPR2 in the Regulation of Epithelial-Mesenchymal Transition

**DOI:** 10.3390/cells9040921

**Published:** 2020-04-10

**Authors:** Padma Murthi, Gayathri Rajaraman, Jan Jaap H.M. Erwich, Evdokia Dimitriadis

**Affiliations:** 1 Department of Obstetrics and Gynaecology, University of Melbourne, Parkville, Victoria 3052, Australia; eva.dimitriadis@unimelb.edu.au; 2 Department of Pharmacology, Monash Biomedicine Discovery Institute, Monash University, Clayton, Victoria 3168, Australia; 3 Hudson Institute of Medical Research, Clayton, Victoria 3168, Australia; 4 First year college, Victoria University, St Albans, Victoria 3021, Australia; gayathri.rajaraman@vu.edu.au; 5 Department of Obstetrics and Gynaecology, University of Groningen, 9700 RB Groningen, The Netherlands; j.j.h.m.erwich@umcg.nl

**Keywords:** small-for-gestation age, placenta, formyl-peptide receptor-2, epithelial-mesenchymal transition, fetal growth restriction

## Abstract

We reported earlier that an anti-inflammatory small peptide receptor-formyl peptide receptor-2 (FPR2) was significantly decreased in placentas from third trimester pregnancies complicated with fetal growth restriction (FGR), compared to placentas from uncomplicated control pregnancies, suggesting FPR2 may play a role in the development of FGR. The aim of this study is to investigate whether the actions of FPR2 alters placental growth process in humans. Accordingly, using small-for-gestation age (SGA) as a proxy for FGR, we hypothesize that FPR2 expression is decreased in first-trimester placentas of women who later manifest FGR, and contributes to aberrant trophoblast function and the development of FGR. Chorionic villus sampling (CVS) tissues were collected at 10–12 weeks gestation in 70 patients with singleton fetuses; surplus tissue was used. Real-time PCR and immunoassays were performed to quantitate FPR2 gene and protein expression. Silencing of FPR2 was performed in two independent, trophoblast-derived cell lines, HTR-8/*SVneo* and JEG-3 to investigate the functional consequences of FPR2 gene downregulation. *FPR2* mRNA relative to *18S rRNA* was significantly decreased in placentae from SGA-pregnancies (*n* = 28) compared with controls (*n* = 52) (*p* < 0.0001). Placental FPR2 protein was significantly decreased in SGA compared with control (*n* = 10 in each group, *p* < 0.05). Proliferative, migratory and invasive potential of the human placental-derived cell lines, HTR-8/*SVneo* and JEG-3 were significantly reduced in *siFPR2* treated cells compared with *siCONT* control groups. Down-stream signaling molecules, *STAT5B* and *SOCS3* were identified as target genes of FPR2 action in the trophoblast-derived cell lines and in SGA and control chorionic villous tissues. FPR2 is a novel regulator of key molecular pathways and functions in placental development, and its decreased expression in women destined to develop FGR reinforces a placental origin of SGA/FGR, and that it contributes to causing the development of SGA/FGR.

## 1. Introduction

Fetal growth restriction (FGR) complicates up to 10% of all human pregnancies and is a major cause of preterm birth and late pregnancy stillbirth, a leading cause of neonatal death and morbidity and a cause of lifelong neurological impairment including cerebral palsy and cardio-metabolic and vascular diseases in adulthood [1,2,3,4]. Small-for-gestation age (SGA) is often considered as a surrogate for FGR. While a number of maternal and fetal factors that contribute to FGR/SGA have been identified, the etiologies of the mechanism has not yet been fully elucidated. The underlying cause of FGR/SGA is unclear, but in the absence of a maternal (e.g., preeclampsia) and/or fetal (e.g., genetic) pathology, the origins of FGR/SGA predominantly lie within a functionally insufficient placenta, which manifests as inadequate utero-placental blood flow on ultrasound scan and maternal vascular mal-perfusion on placental histology [5].

Placental-related FGR/SGA arises primarily due to deficient remodeling of the uterine spiral arteries supplying the placenta during early pregnancy. The resultant mal-perfusion induces cellular stress within the placental tissues, leading to selective suppression of protein synthesis and reduced cell proliferation [6]. Consequently, there is a reduction in villous volume and surface area for maternal-fetal exchange. Extensive dysregulation of gene expression occurs, affecting placental transport, endocrine, metabolic and immune functions. Secondary changes involving dedifferentiation of smooth muscle cells surrounding the fetal arteries within placental stem villi correlate with absent or reversed end-diastolic umbilical artery blood flow, and with a reduction in birthweight [6]. Thus, the developmental abnormalities associated with fetal growth and the clinical symptoms associated with pathological pregnancies in late pregnancy are often associated with abnormal placental development early in gestation [6].

Excessive placental inflammation is associated with several pathological conditions, including FGR pregnancies [7]. We have recently reported that a small molecule peptide receptor, formyl-peptide receptor (FPR2), an anti-inflammatory receptor, is expressed in the human placenta across gestation [8]. FPRs are seven trans-membrane proteins belonging to the G-protein coupled receptor family. In humans, three FPR paralogs have been identified (FPR1, FPR2 and FPR3). Upon binding to specific ligands/agonists, FPR2 influences basic cellular functions including proliferation, differentiation, invasion, angiogenesis as well as host defense and regulation of inflammatory reactions [9]. As such, FPRs and their specific agonists have been identified as potential targets in the development of efficient therapeutic agents in several diseases including cancer, diabetes and asthma [9,10,11].

Targeted deletion of *Fpr2* in mice and/or in vitro silencing leads to increased inflammation, compromised immune regulation and abnormal blood vessel growth, whereas FPR activation or over-expression inhibited inflammation, restored immune regulation and increased neovascularization [12,13]. FPR2 regulates inflammatory events in the human endometrium and decidua of early pregnancy, including production of the pregnancy hormone, β-human chorionic gonadotropin (β-hCG) early in pregnancy [14]. Our previous study also demonstrated that FPR2 expression was significantly decreased in the placentas from third-trimester FGR pregnancies compared with uncomplicated normal pregnancies; and its reduction was associated with an aberrant production of inflammatory cytokines and chemokines from cultured syncytiotrophoblast (SCT) in vitro [8]. However, determining the cause of human FGR remains a major challenge in human pregnancy research. It is uncertain whether the decreased placental FPR2 expression observed in third trimester human FGR-affected pregnancies [8] is truly causative or rather reflects a response to an altered growth process.

A recent study describes that FPR2 plays an essential role in the regulation of epithelial mesenchymal transition (EMT) in tumor progression [15]. However, the potential role of FPR2 in EMT associated with early placentation is largely unknown. Human placental development shares many features of a metastatic tumor biology. More specifically, during early placental development, the extravillous trophoblasts (EVT) undergo a partial EMT and migrate from the outer surface of the cytotrophoblastic shell into the endometrium by adopting a pleiotropic phenotype similar to metastatic tumor tissue [16]. The placenta has therefore been linked to a malignant tumor, albeit a highly regulated one [17]. Understanding the role of key regulators such as FPR2 in processes associated with EMT in the earliest stages of pregnancy is critical to comprehending the role of the FPR2 not only in uncomplicated pregnancies but also in pathological pregnancies associated with abnormal EMT in FGR pregnancies. Therefore, in this study we determined the expression of FPR2 in early placental tissues collected at first trimester and investigated the signaling pathways and EMT that are essential for trophoblast proliferation and invasion.

Early gestation sampling can be accomplished by chorionic villus sampling (CVS), a procedure generally performed between 10 and 14 weeks of gestation. In this study, we made use of a unique resource of first trimester tissues collected via CVS during the first trimester. Subsequent differentiation between uncomplicated and pathological pregnancies is possible, as the eventual maternal and fetal outcomes of ongoing pregnancies are determinable [18,19,20]. We hypothesized that placental FPR2 expression is decreased early in gestation in SGA pregnancies and contribute to aberrant signaling mechanisms associated with trophoblast invasion and EMT. FPR2 expression was quantified using real-time PCR and immunoassays in villus tissue collected with known clinical outcomes, i.e., SGA or uncomplicated control pregnancies, to investigate the temporal relationship between altered placental FPR2 expression and any subsequent development of SGA. The consequences of decreased FPR2 expression on trophoblast function was determined using two independent trophoblast-derived cell lines, HTR-8/*SVneo* and JEG-3.

## 2. Materials and Methods

### 2.1. Human Research Ethics

The collection and archiving of all samples into a biobank for future research purposes had the approval of the relevant institutional human research ethics committees as described below for the CVS and the first trimester placental villus tissues. Each woman, from whom samples were collected and pregnancy outcome data recorded, gave informed and written consent to the collection of the placental villus tissue and the recording of coded, de-identified demographic information and pregnancy outcomes where possible.

### 2.2. Collection of Surplus CVS Tissue Samples

First-trimester placental villous tissue was obtained from surplus tissue at chorionic villus sampling (CVS) from singleton pregnancies performed vaginally between 10 and 12 weeks of gestation for maternal age or serum screening related risk for aneuploidy. CVS was performed at the University Medical Center of Groningen, The Netherlands. Surplus supply of CVS samples was collected from pregnant women with informed consent and in accordance with the guidelines of the Federation of Dutch Medical Scientific Societies regarding surplus material not needed for diagnostics. Patient demographics including follow-up of pregnancy outcome details were collected by a questionnaire returned by the patient postpartum. As previously described in our study [18,19,20], pregnancies later complicated by SGA were selected from the database, and controls were selected matched for maternal age and gestational age at the time of sampling. SGA cases were selected based on the birth weight being below the 10th percentile according to Dutch population charts from the Stichting Perinatale Registratie Nederland. Patient identification was then removed, and the samples were coded and processed anonymously. The exclusion criteria for both control and SGA affected pregnancies were chromosomal abnormalities, congenital anomalies, gestational diabetes, preeclampsia, maternal hypertension and maternal chemical dependency. The control group was selected based on the crown–rump-length (CRL) of each SGA affected fetus at the time of CVS. A total of 105 pregnancies were selected based on the RNA quality, *n* = 28 from SGA and *n* = 52 from control uncomplicated pregnancies were used (Table 1).

### 2.3. First Trimester Placental Tissues

Placental tissues from pregnancies at the first-trimester were obtained with the informed written consent from Monash Health, Clayton, Victoria, Australia. Placental tissues were collected from 10–12 weeks’ gestation (*n* = 3).

### 2.4. Trophoblast-Derived Cell Lines

The immortalized isolated primary normal extravillous cytotrophoblasts (EVCT), HTR-8/*SVneo* was a kind gift from Dr. Charles Graham (Queen’s University, Kingston, ON, Canada). The choriocarcinoma-derived cell line, JEG-3 was purchased from American Tissue Type Culture Collection (ATCC, Manassas, VA, USA). The cells were maintained in RPMI-1640 medium supplemented with 10 mM sodium bicarbonate, 50 mg/mL streptomycin, 50 IU/mL penicillin and 10% fetal bovine serum (FBS). All reagents were purchased from Invitrogen Corporation (Carlsbad, CA, USA).

### 2.5. Silencing of FPR2 in Trophoblast Cell Lines

Cultured HTR-8*/SV-neo* and JEG-3 (2×10^5^ cells/well in 6-well plates and 5×10^4^ cells/well in 24-well plates) cells were transfected with siRNA specific for *FPR2* (*siFPR2,* Thermo Fisher Scientific, Waltham, MA, USA). Negative control siRNA (*siCONT*) consisted of a pool of enzyme-generated siRNA oligonucleotides of 15–19 base pairs that were not specific for any known human gene (AllStars Neg. siRNA AF 488, Qiagen, Hilden, Germany) and showed no sequence similarity to *FPR2.* Briefly, *siFPR2* or *siCONT* were used at a ratio of 1:6 and incubated for 15 min at room temperature. The siRNA complexed with the Hi-Perfect transfection reagent (Qiagen, Hilden, Germany) was then added drop-wise to a final concentration of 80-µM siRNA and incubated at 37 °C for 24–48 h hours. Trypan blue exclusion assay was performed to verify the viability of cells following siRNA transfection.

### 2.6. Real-Time PCR

Total placental RNA was isolated and purified using the Macherey–Nagel NucleoSpin^®^ RNA kit according to the manufacturer’s instructions (Macherey–Nagel Inc. Dueren, Germany). cDNA was prepared from 500 ng total RNA using a Qiagen QuantiTect Reverse Transcription Kit according to the manufacturer’s instructions (Qiagen, Hilden, Germany). *FPR2* mRNA expression was determined using validated assays that consisted of a TaqMan^®^ FAM^TM^ labeled MGB probe (*FPR2*, Hs02759175_s1, Thermo Fisher Scientific, Waltham, MA, USA) on an ABI Prism 7500 (Thermo Fisher Scientific, Waltham, MA, USA). Gene expression quantitation was performed as the second step in a two-step reverse transcriptase–polymerase chain reaction (RT-PCR) protocol according to the manufacturer’s instructions (Invitrogen/Thermo Fisher Scientific, Waltham, MA, USA). Gene expression quantitation for the housekeeping gene *18S rRNA* (VIC- labeled probe, Thermo Fisher Scientific, Waltham, MA, USA) was performed in the same reaction as described previously [8,20]. Levels of gene expression relative to *18S rRNA* were calculated according to the 2^-ΔΔCT^ method [8,20,21].

### 2.7. Immunohistochemistry

Paraffin-embedded 5 µm tissue sections were deparaffinized in xylene and dehydrated in graded alcohol. Endogenous peroxidase activity was blocked using 3% hydrogen peroxide for 10 min at room temperature. Antigen retrieval was achieved by enzymatic digestion using 20 mg/mL Proteinase K (Ambion, Austin, TX, USA) in Tris buffer containing 1 M Tris-HCl, 0.5 M EDTA (pH 7.5). Nonspecific protein binding was saturated with the blocking agent provided in the Histostain-Plus Broad Spectrum kit (Zymed Laboratories, South San Francisco, CA, USA). Tissue sections were then incubated overnight at 4 °C with primary FPR2 anti-human, mouse monoclonal IgG antibody (Novus Biologicals, Centennial, CO, USA) at a concentration of 0.02 µg/µl, in 2% (w/vol) non-fat milk phosphate buffered saline (PBS). Control sections were incubated with 0.02 µg/µl mouse IgG, 2% (w/vol) non-fat milk in PBS (DAKO, Copenhagen, Denmark). Staining was visualized by incubating with the biotinylated secondary antibody and streptavidin-conjugated enzyme from the Histostain-Plus Broad-Spectrum kit (Zymed Laboratories, South San Francisco, CA, USA). Chromogenic detection was performed using diaminobenzidine (DAB, Sigma Chemical Co. St. Louis, MO, USA). Sections were mounted with Histomount (Sigma Chemical Co. St. Louis, MO, USA).

### 2.8. ELISA

FPR2 in the total protein extracts prepared from the chorionic villus tissues (*n* = 10 SGA; *n* = 10 control) and from the cellular extracts were measured using an ELISA (FPR2 ELISA Kit (Human), OKEH01364, Aviva Systems Biology, San Diego, USA) following the manufacturer’s instructions. The total protein extract from CVS tissues were obtained as a second step purification from the Macherey–Nagel NucleoSpin^®^ RNA kit (Macherey–Nagel Inc. Dueren, Germany), due to the limitations on the purity of the protein content, only a subset of CVS samples was analyzed for protein content. The concentration of FPR2 (pg/mL) in μg of total protein in the chorionic villus samples and in the cellular extracts were determined using a microplate reader, SpectraMax i3 (Molecular Devices, San Jose, CA, USA) and the optical density was read at an absorbance of 450 nm.

### 2.9. Proliferation Assay

Cultured HTR-8*/SV-neo* and JEG-3 cells were seeded into 24-well plates at a density of 5 × 10^4^ cells/well, cultured and transfected for 24 h using the *siFPR2* or *siCONT*. Cell proliferation was measured at 48 h using the CyQuant proliferation assay (Thermo Fisher Scientific, Waltham, MA, Australia) and the fluorescence measurements were made using a microplate reader SpectraMax i3 (Molecular Devices, San Jose, CA, USA) with excitation at 485 nm and emission detection at 530 nm.

### 2.10. Transwell Migration and Invasion Assays

Cell migration and invasion in HTR-8*/SV-neo* and JEG-3 were measured using 8 µm tissue culture inserts (Becton and Dickinson, BD Biosciences, San Jose, CA, USA). For the invasion assay, the polycarbonate membranes at the bottom of the Transwell were coated with a thin layer of growth factor-reduced Matrigel (Becton and Dickinson, BD Biosciences, San Jose, CA, USA), diluted 1:10 in RPMI medium. Cell invasion and migration was quantified using colorimetric methods as previously described [22]. Briefly, cells were serum starved overnight prior to *siFPR2* or *siCONT* transfection. Cells were transfected for 48 h, detached using non-enzymatic cell dissociation reagent (Sigma Chemical Co. St. Louis, MO, USA) and plated in the Transwell using serum free medium at 5 × 10^3^ cells/chamber. The lower chambers contained 10% FBS medium as a chemo-attractant. Following incubation for 24 h at 37 °C in 95% air/5% CO_2_, non-migrating cells in the Transwell inserts were removed using a cotton swab. Alamar Blue (Becton and Dickinson, BD Biosciences, San Jose, CA, USA) was added to the medium of the feeder tray at a final concentration of 10% and the plate was further incubated at 37 °C for 8 h. Absorbance was read at 540 and 630 nm with a microplate reader, SpectraMaxi3 (Molecular Devices, San Jose, CA, USA).

### 2.11. Apoptosis Assay

The effect of FPR2 on apoptosis in HTR-8*/SV-neo* and JEG-3 cells was measured following transfection with siFPR2 or siCONT for 48 h in culture. Apoptosis was determined using the ApoAlert^®^ Caspase Colorimetric assay kit specific for the caspase 3 activity according to the manufacturer’s instructions (Clone Tech Laboratories Inc/ Takara Bioscience, Mountainview, CA, USA). The colorimetric assay was based on spectrophotometric detection of the chromophore *p*-nitroaniline (pNA) after its cleavage by caspases from the labeled caspase-specific substrates as previously described [23]. Briefly, cells (2 × 10^5^ cells/well) were treated with *siFPR2* or *siCONT* as described above. ApoAlert Caspase activity for caspase 3 was measured on cell lysates by the addition of 50 μM caspase 3 substrate (DEVD-pNA) in the presence or absence of caspase 3 inhibitor DEVD-fmk, all provided in the assay kit. Chromgen detection and absorbance were measured at 405 nm using a microplate reader, SpectraMaxi3 (Molecular Devices, San Jose, CA, USA).

### 2.12. Immunoblotting for Markers of Apoptosis and EMT

Total cellular protein from HTR-8*/SV-neo* and JEG-3 cells was extracted and markers of apoptosis were quantitated by immunoblotting as previously described [8]. Immunoblotting was performed with 25 μg of total protein using a 10% SDS/PAGE and electroblotting onto a nitrocellulose membrane (Pal Gelman, NSW, Australia). The membrane was blocked with 5% (w/v) skim milk for one hour at room temperature and followed by an overnight incubation at 4 °C in 0.025 μg/μL mouse anti-human monoclonal p53 (TP53 PAb 1801, Abcam, Cambridge, MA, USA) or 0.01 μg/μL mouse anti-human monoclonal Caspase 8 (MAB4708, Chemicon, Australia) or rabbit polyclonal E-cadherin (AB 15148, Abcam, Cambridge, MA, USA) or SNAL (AB82846, Abcam, Cambridge, MA, USA) or SLUG (AB27568, Abcam, Cambridge, MA, USA) or mouse monoclonal vimentin (E5, Santa Cruz Biotechnology, Dallas, Texas, USA) or mouse monoclonal beta-tubulin (loading control, NB600-501, Novus Biologicals, Centennial, CO, USA). Antibody binding was visualized using horseradish peroxidase-conjugated goat anti-mouse secondary antibody (0.02 μg/μL, Thermo Fisher Scientific, Waltham, MA, USA), followed by autoradiography using the ECL-Western Chemiluminescence Detection Kit (GE Healthcare, Chicago, Illinois, USA). Immunoreactive protein for TP53 (53 kDa), Caspase 8 (55 kDa), E-cadherin (120 kDa), SNAIL (30 kDa), SLUG (29 kDa) and vimentin (55 kDa) normalized to beta-tubulin (50 kDa) was quantitated using scanning densitometry (ImageQuant, GE Healthcare, Chicago, Illinois, USA) as described previously [8].

### 2.13. cDNA Array

RNA from three independent transfection experiments from the HTR-8/*SVneo* and JEG-3 cells were pooled separately and a total of 2 µg RNA per PCR array plate was used for each of the treatment groups for the two cell lines. The TaqMan^®^ Array on Human JAK-STAT pathway (catalog # 4414156, Thermo Fisher Scientific, Waltham, MA, USA) and custom-designed EMT markers cDNA array (Thermo Fisher Scientific, Waltham, MA, USA) were performed following cDNA preparation using the Superscript III containing reagents, according to the manufacturer’s recommendations (Thermo Fisher Scientific, Waltham, MA, USA). PCR conditions consisted of an activation cycle at 50 °C for 2 min and 95 °C for 10 min, followed by 40 cycles at 95 °C for 15 s and 60 °C for 1 min. mRNA expression was normalized to the housekeeping gene included in the array plate. Data were analyzed using Data Assist software program (Thermo Fisher Scientific, Waltham, MA, USA). Differentially expressed genes were prioritized based on their level of expression above or below a 2-fold change in mRNA expression in *siFPR2* treated cells compared with *siCONT* treated cells. Complementary genes that showed consistent up-regulation or down-regulation in the two cell lines were further validated by real-time PCR in the cultured cells and in the CVS samples. As described above, gene expression relative to *18S rRNA* was calculated according to the 2^-ΔΔCT^ method [8,20,21].

### 2.14. Data Analysis

Statistical analysis was performed using Graph Pad Prism program (GraphPad software, Version 7.01, Inc., San Diego, CA, USA). Mann–Whitney U test was used for the semi-quantitative data analysis for *FPR2* mRNA, while unpaired *t*-test was used for differences in FPR2 protein between SGA and control groups. Statistical differences between treatment groups were evaluated using paired t-test. All data are presented as ± SEM, unless otherwise stated. A probability value of <0.05 was accepted as statistically significant and 95% confidence intervals (CI) are given where appropriate.

## 3. Results

Table 1 depicts the demographic data collected at delivery for both SGA and control pregnancies used in this study. A significantly lower mean birth weight was observed in the SGA group compared with controls, however, there was no significant difference in maternal age, gestation or parity between the two groups.

**Figure 1:**
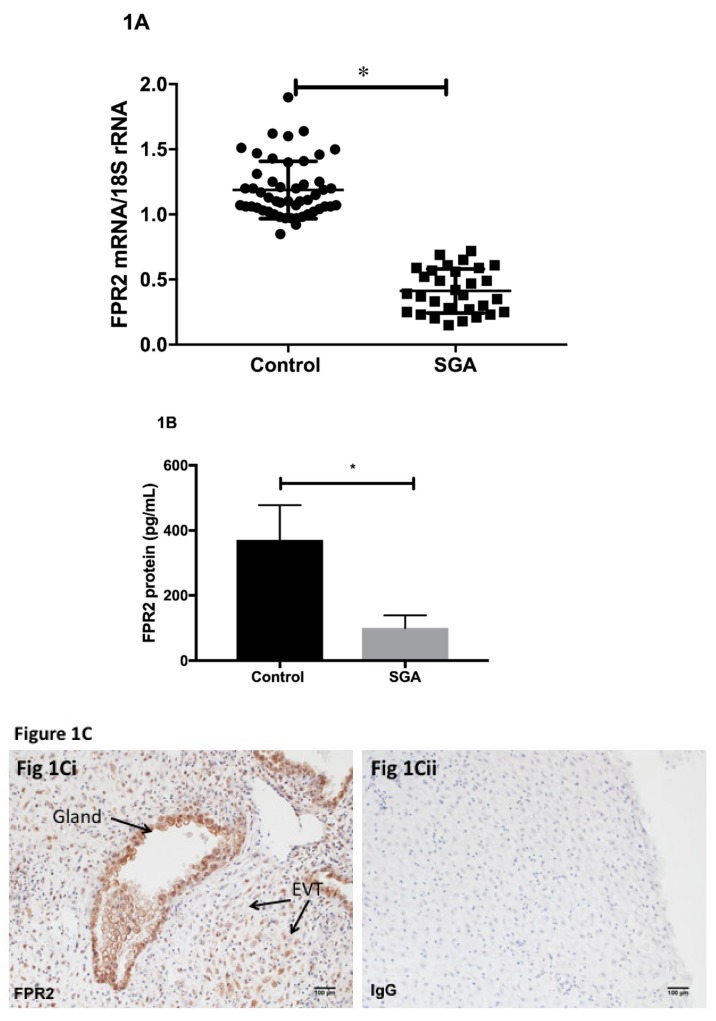
(**A**) Real-time PCR analyses for *formyl peptide receptor-2 (FPR2)* mRNA relative to *18S rRNA.* *FPR2* mRNA relative to *18S rRNA* in SGA (*n* = 28) and control (*n* = 52) in early gestation was determined using real-time PCR. Gene expression differences between SGA and control placental tissues were calculated according to 2^-ΔΔCT^ method (Livak and Schmittgen, 2001). Data presented as mean ± SEM. A probability value of <0.05 was considered to be statistically significant as denoted by *. (**B**) ELISA. FPR2 concentrations in the chorionic villus tissues from SGA (*n* = 10) and control (*n* = 10) pregnancies. Data presented as mean ± SEM. A probability value of <0.05 was considered to be statistically significant as denoted by *. (**C**) Immunohistochemical localization for FPR2 protein. Immunoreactive FPR2 protein localization in placental tissues obtained from pregnancies at first trimester (*n* = 3) was performed using immunohistochemistry. A representative image is shown. Arrows indicate the presence of immunoreactive FPR2 protein in; extravillous cytotrophoblasts (EVCT), in the decidual cells and in the glandular epithelial cells. No immunoreactivity was observed in the negative control, where the primary antibody was substituted with the isotype control IgG2a (Figure 1Cii).

Using placental tissues collected via CVS, we determined the expression of FPR2 mRNA in early pregnancy. Quantification of relative *FPR2* mRNA to *18S rRNA* in the chorionic villus samples was performed using real-time PCR (Figure 1A). *FPR2* mRNA was detected in all CVS samples analyzed. The level of expression of placental *FPR2* mRNA was significantly decreased in SGA-affected pregnancies (*n* = 28) compared with those obtained from control pregnancies (*n* = 52; *p* < 0.001; Mann–Whitney U test). Placental content of FPR2 protein was measured using an ELISA. FPR2 protein determined in a subset of chorionic villus tissues from SGA pregnancies (*n* = 10) was significantly decreased compared with that in a subset of control CVS (*n* = 10, *p* < 0.05) (Figure 1B).

Immunohistochemistry was used to localize FPR2 protein in the first-trimester placental tissues at the implantation site from uncomplicated normal pregnancies. Arrows indicate the presence of immunoreactive protein in extravillous cytotrophoblasts (EVCT), in the decidual cells and in the glandular epithelial cells (Figure 1Ci). Substitution of the primary antibody with the isotype control IgG2a (Figure 1Cii) showed no immunoreactivity (Figure 1Cii).

**Figure 2:**
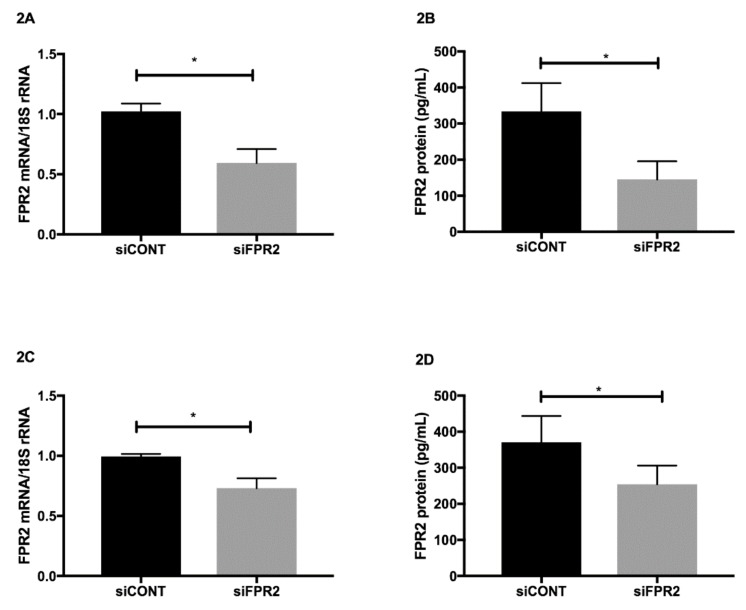
*FPR2* inactivation in HTR-8/*SVneo* and JEG-3. Real-time PCR and ELISA demonstrates mRNA and protein concentrations following transfection with *siFPR2* or *siCONT*. (**A**) (HTR-8/*SV neo*) and (**C**) (JEG-3). Columns represent 2^–ΔΔCT^ values normalized to *18S* mRNA expression, ± SEM of four independent experiments (* *p* < 0.05). (**B**) (HTR-8/*SV neo*) and (**D**) (JEG-3): ELISA to quantitate protein concentration in the cellular extracts of siFPR2 and siCONT treated cells. Data are expressed as the optical density read at an absorbance of 450 nm. Values represent ± SEM of four independent experiments (* *p* < 0.05).

*FPR2 mRNA* in trophoblast-derived cell lines was transiently inactivated using FPR2 specific *siFPR2*. Non-specific siRNA control *siCONT,* was used as a control. In both HTR-8*/SVneo* (Figure 2A) and in JEG-3 (Figure 2C), *siFPR2* treatment for 48 h significantly decreased *FPR2* mRNA compared with cells transfected with *siCONT*, respectively (*n* = 4, *p* < 0.01). The decrease in *FPR2* mRNA expression was further confirmed at the protein level. A quantitative decrease in FPR2 protein was observed in the cellular extracts of both HTR-8/*SVneo* (Figure 2B) and JEG-3 (Figure 2D) cells following *siFPR2* treatment compared with *siCONT* treated cells respectively (*n* = 4, *p* < 0.01).

**Figure 3:**
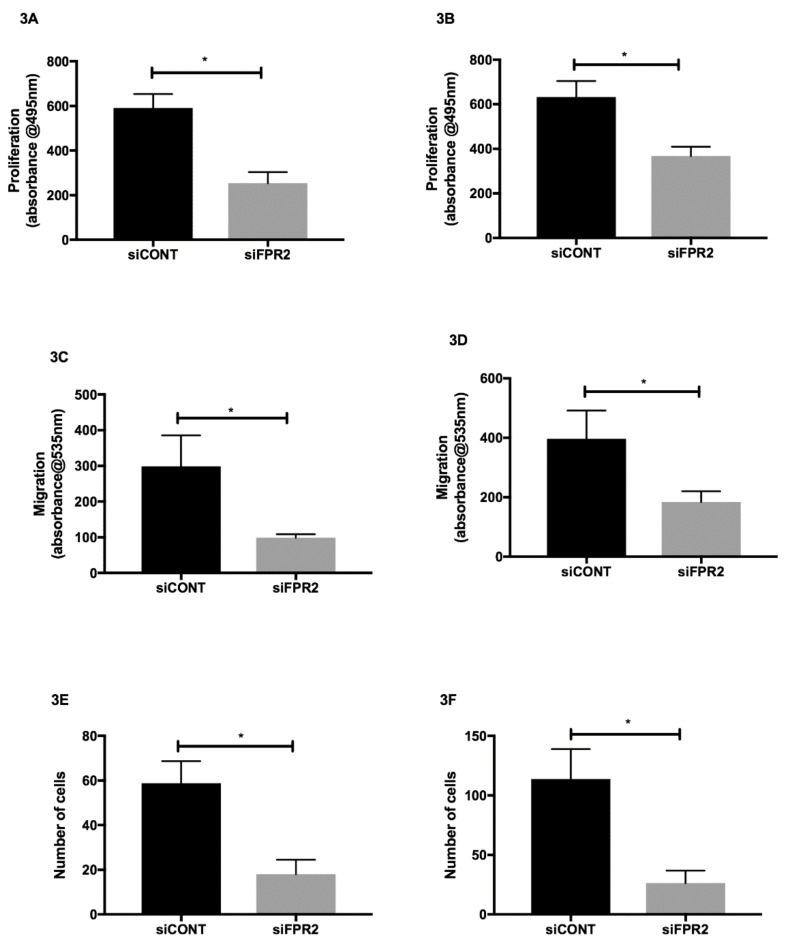
Effect of *FPR2 silencing* on (**A**)**.** (HTR-8/*SV neo*) and (**B**)**.** (JEG-3) proliferation; (**C**) (HTR-8/*SV neo*) and (**D**) (JEG-3) Migration; and (**E**) (HTR-8/*SV neo*) and (**F**) (JEG-3) invasion. Cells transfected for 24 h with siCONT and siFPR2 for proliferation assays. Cells were transfected for 48 h to determine the migratory and invasive potential of FPR2. Briefly, cells were dissociated and seeded on top of uncoated (for migration assay) or Matrigel-coated (for invasion assay) 8-µm pore filters, immersed in feeder trays. The number of migrating and invading cells were deduced according to Al Naisry et al. (2007). Data are representative of four independent experiments conducted in quadruplicate. Values represent ± SEM (* *p* < 0.05).

The effect of *FPR2* silencing on cell proliferation, migration and invasion was also determined in HTR-8/*SVneo* and in JEG-3 cells. Following *siFPR2* treatment for 24 h, the proliferative potential was significantly decreased in both HTR-8/*SVneo* (Figure 3A) and in JEG-3 (Figure 3B) compared with *siCONT* treated cells respectively. Following *siFPR2* transfection for 48 h the percentage of migrated cells or invading cells was calculated and revealed a significant decrease in migratory potential in HTR-8/*SVneo* compared with siCONT treated cells (Figure 3C, *n* = 4, *p* < 0.005) and JEG-3 cells compared with siCONT treated cells (Figure 3D, *n* = 4, *p* < 0.005). *siFPR2* treatment in both HTR-8/*SVneo* (Figure 3E) and in JEG-3 (Figure 3F) resulted in a significant reduction in cell invasion compared with cells transfected with siCONT respectively (*n* = 4, *p* < 0.005).

**Figure 4:**
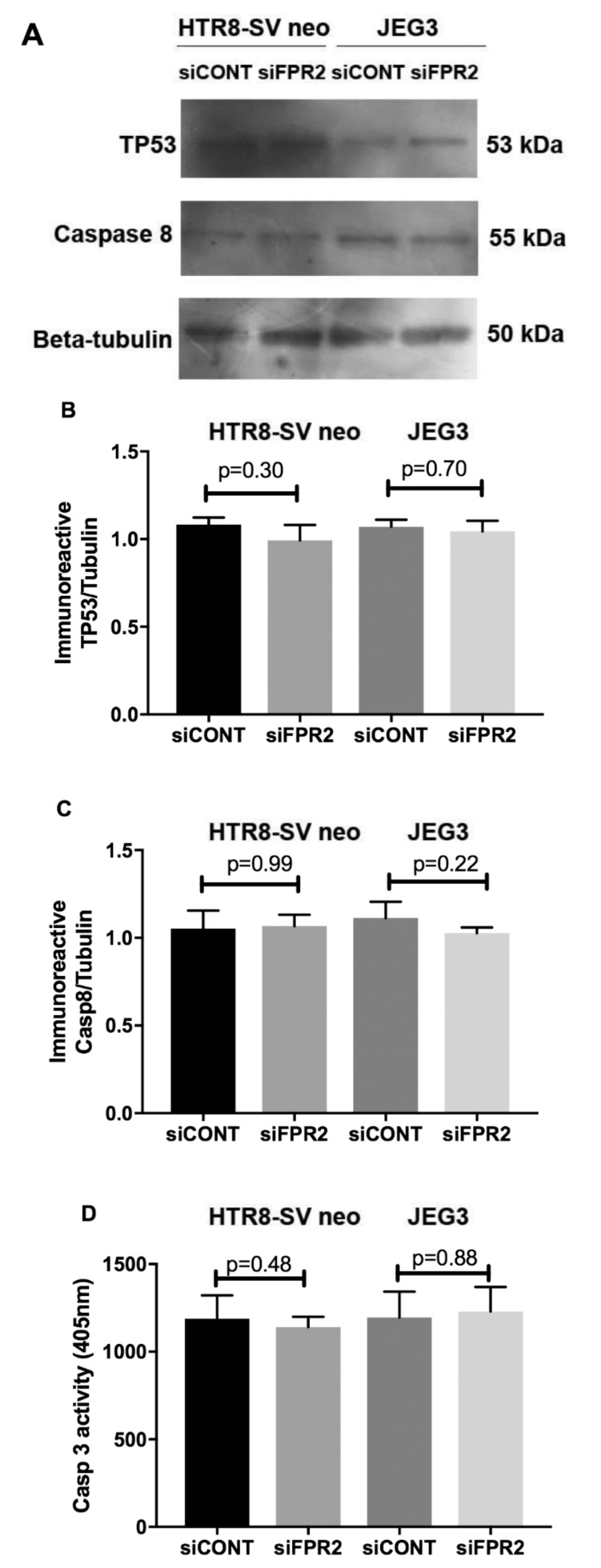
Effect of *FPR2 silencing* on HTR-8/*SV neo* and JEG-3 apoptosis. (**A**)**.** Immunoblotting for TP53, caspase 8 and beta-tubulin loading control in HTR-8/*SV neo* and JEG-3; (**B**)**.** Semi-quantitation of immunoreactive TP53/beta-tubulin in HTR-8/SV *neo* and JEG-3. (**C**)**.** Semi-quantitation of immunoreactive protein caspase 8/beta-tubulin in HTR-8/SV neo and JEG-3. (**D**)**.** Measurement of caspase 3 activity in HTR-8/SV neo and JEG-3. Briefly, cells transfected for 48 h to determine the effect of FPR2 silencing on apoptosis. Data are representative of three independent experiments conducted in duplicate. Values represent ± SEM.

As depicted in Figure 4, the functional effect of FPR2 on HTR8/*SVneo* and in JEG-3 cell apoptosis was investigated following siFPR2 silencing. As shown in Figure 4A, immunoblotting for apoptotic markers detected the presence of immunoreactive proteins TP53 (55 kDa) and caspase 8 (53 kDa) in both HTR-8/*SVneo* and in JEG-3 following *siFPR2* or *siCONT* treatment for 48 h in culture. Semi-quantitative analyses of TP53 (Figure 4B) and caspase 8 (Figure 4C) relative to beta-tubulin (50 kDa) did not show significant difference for both TP53 and caspase 8 in HTR-8/*SVneo* and in JEG-3 cells respectively. Further quantitation of caspase 3 activity in both HTR-8/*SVneo* and in JEG-3 following *siFPR2* or *siCONT* treatment for 48 h in culture, also did not show a significant difference in *siFPR2* treated cells compared to *siCONT* (Figure 4D).

**Figure 5:**
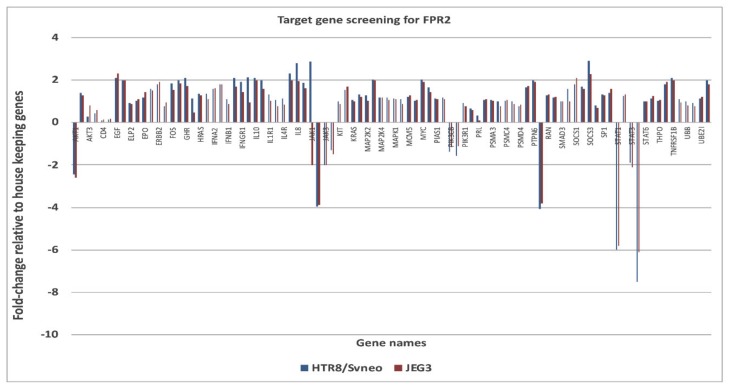
Relative mRNA expression for all target genes in the JAK-STAT signaling pathway as described in the methods section. Columns represent 2^–ΔΔCT^ values, normalized to three house-keeping controls. The data represent pooled (*n* = 4 independent experiments) cDNA collected from *siFPR2 or siCONT* treated HTR-8/SV neo (red) and JEG-3 (blue). Further validation of the candidate genes was performed using the real-time PCR as described in the methods section.

The down-stream targets of genes of FPR2 in the JAK-STAT pathway was investigated using a pathway specific cDNA array. The candidate genes identified and prioritized for those that showed an increase (>2-fold) or decrease (<2-fold) expression changes (Figure 5). Genes that were complementary in both the cell lines and showed consistent gene expression differences between treatment groups were identified as *AKT1* ( − 2.45 in HTR-8/*SVneo* and −2.60 in JEG-3); *RAF1* (−4.08 in HTR-8/*SVneo* and −3.81 JEG-3); *JAK2* (−3.95 in HTR-8/*SVneo* and -3.90 in JEG-3); *STAT1* (−6.0 in HTR-8/*SVneo* and −5.8 in JEG-3); *STAT5B* (−7.5 in HTR-8/*SVneo* and −6.1 in JEG-3). An increase mRNA expression for suppressor of cytokine signaling 3 (SOCS3) in HTR-8/*SVneo* (+2.9) and in JEG-3 (+2.3) was observed.

**Figure 6:**
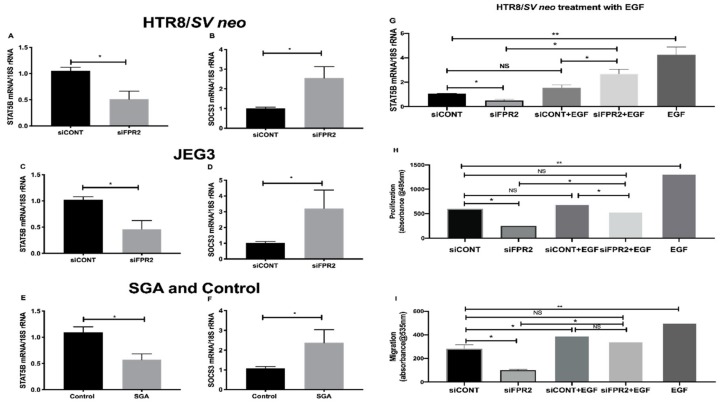
Relative quantification of mRNA of the candidate *FPR2* downstream targets in HTR-8/*SVneo*, JEG-3 and in SGA and control chorionic villus tissues by real time PCR. (**A**) and (**B**) in HTR-8/*SV neo***:** Relative mRNA expression for *STAT5B* and *SOCS3*. (**C**) and (**D**) in JEG3: Relative mRNA expression for *STAT5B* and *SOCS3*. (**E**) and (**F**) in SGA and Control chorionic villus tissues: Relative mRNA expression for *STAT5B* and *SOCS3*. Columns represent 2^–ΔΔCT^ values, normalized to 18S rRNA, ± SEM are shown (* *p* < 0.05). (**G–I**) in HTR-8/*SVneo*, EGF (10 ng/mL) stimulation following siFPR2 or siCONT treatment significantly increased STAT5B mRNA relative to *18S rRNA* in siFPR2 treated cells compared to siCONT (* *p* < 0.05); (**H**) and (**I**) EGF stimulation (10 ng/mL) significantly improved proliferation and migration of HTR-8/*SVneo* cells following treatment with siFPR2 compared to siCONT treated cells (* *p* < 0.05 and ** *p < 0.005*), respectively. Data are representative of four independent experiments conducted in duplicate. Values represent ± SEM.

Further validation of the candidate genes that consistently showed the most down- or up-regulation in the *siFPR2* treated cells compared with *siCONT* treated cells in both HTR-8/*SVneo* and in JEG-3; and in CVS tissues is shown in Figure 6. A significant decrease in the mRNA of *STAT5B* relative to *18S rRNA* in *siFPR2* treated cells compared with *siCONT* treated cells was observed in HTR-8/*SVneo* (Figure 6A), while the most up-regulated gene, *SOCS3* demonstrated a significantly increased *SOCS3* mRNA in *siFPR2* treated cells compared with *siCONT* treated cells in HTR-8/*SVneo* (Figure 6B). In JEG-3 cells a similar trend for the mRNA following siFPR2 was observed, demonstrating a significant decrease in the mRNA of *STAT5B* (Figure 6C) and an increase in the mRNA of *SOCS3* (Figure 6D) in siFPR2 compared with siCONT treated cells. These candidate genes were further validated in the chorionic villus tissues (Figure 6E,F) and consistent significant differences in *STAT5B* and *SOCS3* mRNA relative to *18S rRNA* expression were observed in the SGA group (*n* = 28) compared with control (*n* = 52).

Further investigation on the direct involvement of FPR2 on STAT5B expression, following siFPR2 or siCONT treatment of HTR-8/SVneo cells for 48 h in culture, cells were stimulated with epidermal growth factor (EGF, 10 ng/mL) for 24 h and STAT5B mRNA expression and functional analysis for proliferation and migration were performed. EGF was chosen for this experiment as FPR2 is implicated in the activation of the phosphorylation of tyrosine residues in the JAK/STAT signaling pathway through EGF-receptor [24]. As shown in Figure 6G–I, EGF stimulation of siFPR2 treated cells showed a significant increase in STAT5B mRNA expression relative to *18S rRNA* (Figure 6G). Furthermore, EGF stimulation of siFPR2 treated cells rescued proliferation (Figure 6H) and migration (Figure 6I) potential of HTR-8/*SVneo* cells compared to siCONT treated cells.

**Figure 7:**
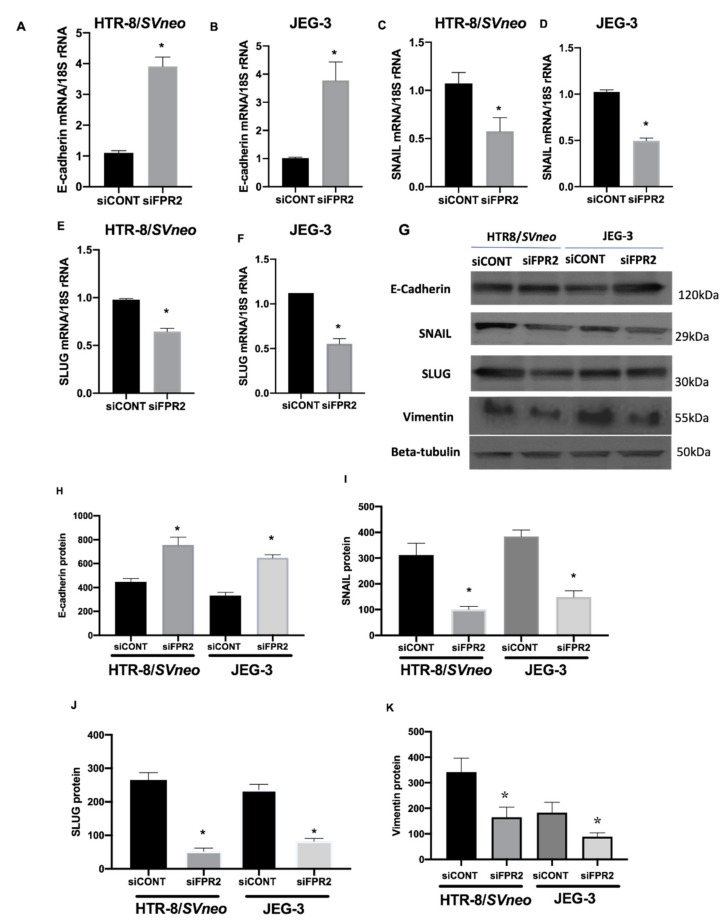
(**A–F**) Relative mRNA expression for all target genes in the epithelial mesenchymal transition (EMT) pathway as described in the methods section. Columns represent mRNA expression normalized to *18S rRNA*. The data represent pooled (*n* = 4 independent experiments) cDNA collected from *siFPR2 or siCONT* treated HTR-8/*SVneo* (black) and JEG-3 (gray). (**G**) Immunoblotting for E-cadherin, SNAIL, SLUG, vimentin and beta-tubulin loading control in HTR-8/*SV neo* and JEG-3; (**H**) Semi-quantitation of immunoreactive E-cadherin/beta-tubulin in HTR-8/SV *neo* and JEG-3. (**I**) Semi-quantitation of immunoreactive protein SNAIL/beta-tubulin in HTR-8/SV neo and JEG-3. (**J**) Semi-quantitation of immunoreactive protein SLUG/beta-tubulin in HTR-8/SV neo and JEG-3. (**K**) Semi-quantitation of immunoreactive protein vimentin/beta-tubulin in HTR-8/SV neo and JEG-3. Briefly, cells transfected for 48 h to determine the effect of FPR2 silencing on markers of EMT. Data are representative of four independent experiments conducted in duplicate. Values represent ± SEM. * *p < 0.05*.

Investigation on the effect of siFPR2 on the downstream target genes in the EMT pathway Candidate genes that showed 2-fold differences in gene expression were prioritized. A 3-fold increase in E-cadherin mRNA was observed in *siFPR2* treated cells in HTR-8/*SVneo,* while 3.7-fold increase in JEG-3 cells compared with *siCONT* treated cells. However, a 2-fold decrease in the mRNA for SNAIL and SLUG was observed in both HTR-8/*SVneo* and JEG-3 cells following *siFPR2* transfection when compared with *siCONT* treated cells respectively. In addition to these EMT markers, vimentin, is an intermediate filament protein which is characteristically upregulated in cells undergoing EMT was also analyzed. Further validation of the EMT target genes by real-time PCR showed a significant increase in E-cadherin mRNA (Figure 7A, HTR-8/*SVneo*; Figure 7B, JEG-3), while a significant decrease in *SNAIL* (Figure 7C, HTR-8/*SVneo*; Figure 7D, JEG-3) and *SLUG* (Figure 7E, HTR-8/*SVneo*; Figure 7F, JEG-3) mRNA following *siFPR2* treatment compared with *siCONT*. Vimentin mRNA expression was 2.5-fold (HRT-8/*SVneo*) and 2.3-fold (JEG-3) decreased in siFPR2 treated cells compared with siCONT treated cells (data not shown).

As shown in Figure 7G, immunoblotting for candidate EMT markers detected the presence of immunoreactive proteins E-cadherin (120 kDa), SNAIL (29 kDa), SLUG (30 kDa) and vimentin (55 kDa) in both HTR-8/*SVneo* and in JEG-3 following *siFPR2* or *siCONT* treatment for 48 h in culture. Semi-quantitative analyses of E-cadherin (Figure 7H), SNAIL (Figure 7I), SLUG (Figure 7J) and vimentin (Figure 7K) relative to beta-tubulin (50 kDa) showed significant increase in E-cadherin while a significant decrease in SNAIL, SLUG and vimentin immunoreactivity in HTR-8/*SVneo* and in JEG-3, respectively.

## 4. Discussion

The molecular basis of fetal growth and development is complex. The results of this current study show that *FPR2* is expressed in early gestation chorionic villus samples and its expression is significantly reduced in pregnancies that later developed SGA. These findings agree with our previous study that demonstrated a significant reduction in placental *FPR2* from human pregnancies complicated by FGR collected at third trimester gestation compared with gestation-matched controls [8]. The third trimester placental samples were collected from a well-defined cohort of idiopathic FGR pregnancies that were carefully selected using strict clinical criteria indicative of placental insufficiency and underlying pathology [8]. The results from this study support a temporal relationship between altered *FPR2* expression and SGA and are consistent with a causal role for placental *FPR2* in the success of pregnancy outcome.

The relationship between abnormal placental development and FGR is complex. Feto–placental growth and functional efficiency early in gestation are orchestrated by a cascade of signaling pathways governed by an array of transcription factors, cytokines, endocrine regulators and growth factors and their receptors [6]. Typically, the placentae in SGA/FGR are smaller than their gestation age-matched controls and they show obvious morphologic defects [6,18,20]. Macroscopic placental lesions are frequently evident, while microscopic defects such as reduced trophoblast proliferation and abnormal villous vasculature with shorter, less branched terminal villi are also observed. Another significant functional defect is utero-placental ischemia due to failure of the EVCTs to effectively carry out the critical processes of invasion, transformation and remodeling of the spiral arteries in the maternal decidua [6]. Differentiation of cytotrophoblasts is fundamental to normal human placental development. In particular, modification of the maternal vessels by EVCTs, which replace the maternal endothelium, is critical for the successful progression of pregnancy, since reduced invasion of interstitial and endovascular cytotrophoblasts is associated with FGR and preeclampsia. Our findings demonstrated that FPR2 may directly or indirectly regulate trophoblast proliferation, migration and invasion of HTR-8/*SVneo* and JEG-3 cells, commonly used cell line models to investigate human placental cell function, raising a potential mechanism by which decreased placental *FPR2* expression observed early in gestation in SGA/FGR may contribute to abnormal feto-placental growth early in gestation, thus contributing to the etiology of SGA/FGR.

Regulation of the key events, including proliferation, migration and invasion are essential for successful placentation and pregnancy outcome [6]. Xu and co-workers [13] have reported that placental lipoxin A4 expression is reduced in preeclamptic pregnancies and in the same study, the authors have demonstrated that administration of a synthetic antagonist of Fpr2 in a rodent model caused abnormal placentation and abortion, which emphasizes the critical importance of *FPR2* in placentation. Our findings in this study using the in vitro human cell culture models demonstrate that the down-regulation of *FPR2* in trophoblast-derived cell lines perturbed not only the proliferatory, migratory ability of the trophoblasts, but also the invasive potential of the trophoblasts, which demonstrates that *FPR2* is necessary for the regulation of basic cellular functions likely early in gestation. Furthermore, in vitro experiment using HTR-8/*SVneo* and JEG-3 demonstrated that there were no significant differences observed in the expression levels of markers of apoptosis following silencing of FPR2. This excludes the possibility that the observed reduced proliferation, migration and invasion in the trophoblast-derived cell lines are not part/consequence of apoptosis following downregulation of FPR2.

FPR2 mediates signaling via G-proteins, which trigger several agonist-dependent signal transduction pathways, including JAK-STAT signaling pathway that are essential for multiple biologic processes including cell proliferation, death, differentiation, migration and invasion [9]. Therefore, we subsequently aimed at identifying the targeting signaling molecules downstream of FPR2 that may directly or indirectly regulate basic cellular functions of the trophoblasts. More specifically, silencing of *FPR2* in vitro identified decreased expression of *STAT5B*, while an increased expression for *SOCS3*, which was consistent with the gene expression differences in the chorionic villous tissues collected from pregnancies affected with SGA/FGR compared with control. *STAT5B* plays an essential role in trophoblast biology by regulating the critical events that are fundamental for successful placentation and fetal growth. It is reported that epidermal growth factor (EGF)-mediated *STAT5B* activation increases cell proliferation, migration and invasion [25]. Furthermore, the JAK-STAT signaling pathway is regulated by a vast array of intrinsic stimuli, including EGF, EGF-receptor (EGFR) and FPR2, which can add plasticity to the response of a cell. The cross talk between FPR2 and EGF receptor (EGFR) is an essential first step in activation of the phosphorylation of tyrosine residues in the JAK/STAT signaling pathway [24]. In our experiments, EGF stimulation of FPR2 knock-down in HTR-8/*SVneo* cells not only demonstrated a significant increase in STAT5B mRNA but also showed a rescue effect on the proliferation and migration potential in HTR-8/*SVneo* cells. Thus, FPR2 plays an eminent role in providing docking sites for recruitment and triggering of critical regulatory molecules associated with EGFR-mediated pathway, which involves JAK-STAT signaling molecules that are essential for cellular growth [9,26]. Future studies could determine the activation of this important pathway in the development of SGA/FGR.

Another important down-stream target of FPR2 that was identified in this study was the suppressor of cytokine signaling 3, commonly known as SOCS3. The significant functional role of cytokine signaling in the regulation of a variety of aspects of cell growth and differentiation predominantly occur through their interaction with receptors of the cytokine receptor superfamily, JAKs and, by the activation of members of the STATs [27]. SOCS proteins on the other hand are characterized by the presence of an SH2 domain and a conserved motif termed the SOCS box [28]. They suppress cytokine signaling through interaction of the SH2 domain with sites of tyrosine phosphorylation on receptors or on JAKs. These interactions either compete for the recruitment of other signaling proteins, directly block the catalytic activity of the kinases and/or target the proteins for degradation through recruitment of ubiquitylation complexes through the *SOCS* box [28]. Although the key regulatory mechanisms by which *SOCS3* contribute to trophoblast function in the human placental biology is unknown, findings from cytokine biology in trophoblasts have reported *SOCS3* as crucial in regulating the optimum levels and functions of the important cytokine leukemia inhibitory factor (LIF) and its receptor LIFR [29,30]. *SOCS3* is known as a physiological antagonist for LIFR-mediated trophoblast differentiation and invasion [30]. The invasive capacities of trophoblasts are positively and negatively regulated by LIF/LIFR signaling on the trophoblast cells by inducing expression of invasion relevant genes, including *STAT3*. *SOCS3* deletion leads to embryonic lethality in murine models, while its overexpression suppresses responses to a number of cytokines including LIF [31]. In this study, we have demonstrated that the expression of *SOCS3* is significantly increased in chorionic villus tissue obtained from SGA compared with control. In cultured monocytes, activation of *FPR2* by specific agonist annexin-A1, initiates depletion of *SOCS3* expression to trigger anti-inflammatory responses [32]. One plausible mechanistic pathway for increased *SOCS3* expression in SGA/FGR chorionic villus tissue may include a direct or indirect regulation of FPR2 on the expression of *SOCS3* in HTR-8/*SVneo* and in JEG-3 cells, however, this mechanistic pathway in the trophoblasts warrants further investigation.

There are several pathways shared between placenta and cancer cells at molecular level. These pathways regulate hyperproliferation, invasion, angiogenesis and immunoevasion. Both cancer cells and trophoblast cells promote migration through activation of epithelial-mesenchymal transition (EMT), which leads to loss of cell-to-cell contact inhibitions [33]. However, the migratory and invasive capacities of invading trophoblasts are spatially and time regulated. These capacities enable either the accomplishment of successful embryo implantation and pregnancy progression when kept under control or the achievement of neoplastic and malignant transformation when such capacities are no longer kept under control. In this study, we have identified an increased expression of E-cadherin, while the transcriptional regulators SNAIL and SLUG were decreased in both trophoblast-derived cell lines. These observations suggest that FPR2 is essential for the progression of EMT during placental development. A recent study has reported that the levels of FPR2 expression is directly correlated increased proliferation and invasion during gastric cancer (GC) progression and with the patients’ overall survival [15]. Using in vitro model systems, the functional role of FPR2 was linked with the regulation of invasion and metastasis of GC cells via MAPK/ERK signaling pathways and in the direct regulation of EMT.

In summary, our study reports that reduced FPR2 expression may directly or indirectly contribute to trophoblast dysfunction and subsequent abnormal spiral arteriole remodeling associated with SGA/FGR pregnancies. In our previous study we also reported that decreased placental *FPR2* may lead to disrupted vascular endothelial cell function in vitro including abnormal angiogenesis and increased endothelial permeability, which are characteristics of FGR placentae [8]. It remains uncertain whether these changes also correlate with the reduced placental FPR2 expression observed in early gestation SGA pregnancies. However, it is plausible that the contribution of FPR2 in the many pathological processes leading to SGA/FGR may not be restricted to alterations in trophoblast dysfunction and endothelial function, since FPR2 has been shown to modulate inflammatory cytokine and chemokine production. This raises yet another possibility that reduced placental FPR2 expression, as an anti-inflammatory receptor protein, may impact directly with cytokine and chemokine signaling pathways that are crucial for feto-placental growth early in gestation and hence fetal growth. Thus, FPR2 may have gestation dependent roles in human placental development. In early gestation, FPR2 may regulate cytotrophoblast functions, including villous and EVCT differentiation. Late in gestation, FPR2 may reduce inflammation induced pathogenesis and switch to stabilize vascular functions, which are critical for fetal growth. Overall, our study reports that altered placental FPR2 and poor fetal growth are both the result from the underlying cause of poor placentation/fetal growth.

## 5. Limitations

Here we used SGA as a proxy for FGR, which provided a rare window into pregnancy disorders such as FGR and to early normal placentation. However, current clinical tools including ultrasound methods do not reliably identify those SGA fetuses with increased risk of morbidity. Thus, SGA may dilute a possible effect, nevertheless our data still show reduced FPR2 expression and its association with SGA pregnancies. It must be mentioned, however, that CVS is the only method to directly access first trimester placental molecular profile in the context of known pregnancy outcomes. The indication for CVS, advanced maternal age, may limit the present findings for younger women. Our samples were from a homogeneous racial group, consistent with the population undergoing CVS at the clinical site, which may limit the findings for all pregnant women. The procedure is not offered to women without risk factors because CVS is associated with risk, e.g., 0.33% pregnancy loss. In conclusion, our results directly support the concept of the placental origins of FGR [6] and allow for targeted investigation of placental derived biomarkers in *early* pregnancy.

Another important limitation of our study is the use of trophoblast-derived cell lines including normal extravillous trophoblast-derived cell line, HTR-8/*SVneo* and the choriocarcinoma derived cell line, JEG-3. The necessity to use such cell lines is based on the fact that isolated primary trophoblast no longer proliferates in culture. Thus, only short-term cultures can be performed with primary cells. Therefore, respective cell lines have been developed to overcome the handicap of missing proliferation of primary trophoblasts in culture. We acknowledge that results obtained from studies using non-primary cells need to be taken with caution, especially when trying to extrapolate such results to the in vivo situation. While trophoblast-derived cell lines have their disadvantages compared to primary cells, they are advantageous in terms of reproducibility, stability and proliferation and thus enable to evaluate the net effect of treatments on mRNA/protein expression.

## Figures and Tables

**Table 1:** Patient characteristics of first trimester small-for-gestation age (small-gestation age, SGA) and control pregnancies.

	Control (*n* = 52)	SGA (*n* = 28)	*p* Value
Maternal Age (years)	38.35 ± 3.14	37.96 ± 4.25	*p* = 0.64
Gravidity	3.14 ± 0.22	3.14 ± 0.32	*p* = 0.97
Parity	1.42 ± 0.13	1.43 ± 0.25	*p* = 0.98
Birth weight (g)	3534 ± 251.4	2575 ± 548.2	
*Birth weight percentile:*			*p* < 0.05 *
*<3rd*			
*3^rd^–4th*		32.14%	
*5th–9th*		39.29%	
*>10th*	100%	28.57%	
Newborn gender:			
*Male*	59.62%	53.57%	*p* = 0.60
*Female*	40.38%	46.43%	

Patient characteristics of first trimester SGA and control pregnancies. Results presented as mean ± SD or *n* (%) as appropriate. Unpaired independent t-test and chi-square test used to calculate *p*-values of continuous and discrete non-parametric data respectively. *p* < 0.05 (indicated by *) considered statistically significant.
